# Risk of complications among diabetics self-reporting oral health status in Canada: A population-based cohort study

**DOI:** 10.1371/journal.pone.0218056

**Published:** 2020-01-09

**Authors:** Kamini Kaura Parbhakar, Laura C. Rosella, Sonica Singhal, Carlos R. Quiñonez

**Affiliations:** 1 Dental Public Health, Faculty of Dentistry, University of Toronto, Toronto, Ontario, Canada; 2 Division of Epidemiology, Dalla Lana School of Public Health, University of Toronto, Toronto, Ontario, Canada; 3 Institute for Clinical Evaluative Sciences, Toronto, Ontario, Canada; 4 Public Health Ontario Toronto, Ontario, Canada; Graduate School of Public Health and Health Policy, City University of New York, UNITED STATES

## Abstract

**Background:**

Periodontitis has been associated with diabetes and poor health. While clear associations have been identified for the diabetes–oral health link, less is known about the implications of poor oral health status for incident complications of diabetes. This study investigated the risk of diabetes complications associated with self-reported “poor to fair” and “good to excellent” oral health among diabetics living in Ontario, Canada.

**Methods:**

This was a cohort study of diabetics who took part in the Canadian Community Health Survey (2003 and 2007–08). Self-reported oral health was linked to electronic health records held at the Institute for Clinical Evaluative Sciences. Participants aged 40 years and over, who self-reported oral health status in linked databases were included (N = 5,183). Cox proportional hazard models were constructed to determine the risk of diabetes complications. Participants who did not experience any complications were censored. Models were adjusted for age and sex, followed by social characteristics and behavioural factors. The population attributable risk of diabetes complications was calculated using fully adjusted hazard ratios.

**Results:**

Diabetes complications differed by self-reported oral health; 35% of the total sample experienced a complication and 34% of those reporting “good to excellent” oral health (n = 4090) experienced a complication in comparison to 38% of those with “fair to poor” oral health (n = 1093). For those reporting “poor to fair” oral health, the hazard of a diabetes complication was 30% greater (HR 1.29; 95% CI: 1.03, 1.61) than those reporting “good to excellent” oral health. The population level risk of complications attributable to oral health was 5.2% (95% CI: 0.67, 8.74).

**Conclusions:**

Our findings indicate that reporting “poor to fair” oral health status may be attributed to health complications among diabetics, after adjusting for a wide range of confounders. This has important public health implications for diabetics in Ontario, Canada.

## Introduction

Diabetes is one of the most prevalent chronic conditions and the sixth leading cause of mortality in Canada [1,2]. Considered an epidemic, the prevalence of the condition continues to grow as an increasing number of Canadians are living with diabetes and prediabetes, and it is expected to affect 13.3 million individuals by 2029 [3]. Although an abundance of research has focused on diabetes control and management of its complications, including cardiovascular disease, renal disease, retinopathy and amputation, less attention has been given to periodontal disease, the sixth most common complication of diabetes [4].

Periodontal disease is a dysbiotic inflammatory condition that destroys the bone and connective tissue supporting teeth. Recent meta-analyses have reported that mild to moderate periodontal disease affects a majority of adults and that approximately 5–20% of any population suffers from its severest form [5–7]. Mild periodontal disease is characterized as an early manifestation of the condition and severe periodontal disease occurs as the result of a chronic state of disease. Approximately 6% of the general Canadian adult population have severe periodontal disease [8]. However, approximately 18% of Canadian diabetics have moderate to severe periodontal disease, making periodontal disease an important concern among diabetics [9].

Both diabetes and periodontal disease drive inflammatory pathology at local and distant sites and many studies have presented evidence for the complex relationship between these two chronic conditions [7,10]. The low-grade immune response that develops from periodontal infection can lead to systemic levels of inflammation, exaggerating the immune-inflammatory response that is instrumental to insulin resistance, thereby increasing the severity of diabetes [11–15]. It is argued that this host-mediated response is the basis for the bidirectional relationship between these chronic diseases.

Evidence for the bidirectional link between periodontal disease and diabetes has received considerable attention, largely on the basis of many cross-sectional studies. However, the implications of periodontal disease and oral health for diabetics in Canada is unknown and there is a lack of longitudinal evidence. A 2015 systematic review concluded that periodontal therapy can reduce blood sugar levels following care, thereby improving metabolic control among diabetics [4]. An earlier cohort study found that study participants who received periodontal therapy experienced a reduction in the risk of diabetes complications and medical costs [16]. Similar studies conducted from 2008–2016 reported reduced pharmaceutical costs, greater immediate and long-term medical cost savings, as well as reduced hospital admissions and physician visits among individuals receiving periodontal treatment [17–19]. As both the prevalence of diabetes and its associated indirect and direct costs continue to grow worldwide [20,21], periodontal therapy may be one solution to improving metabolic control among diabetics. Population level interventions addressing oral health and diabetes could also improve the health of Canadians.

However, there is a paucity of population-level evidence on the association between oral health and diabetes complications in Canada. Much of the recent literature has involved studies conducted in clinical settings and in cohorts that were followed for varying periods rendering findings of low to medium quality [4]. Systematic reviews have also reported mixed findings, warranting the need for further research. This study aims to identify the risk of diabetes complications among a cohort of diabetics reporting “good to excellent” versus “poor to fair” oral health in Ontario, Canada’s most populated province.

## Research design and methods

### Study population

This cohort study was designed with the objective of assessing the risk of diabetes complications among diabetics self-reporting oral health status. The base cohort comprised of Ontario residents who participated in the 2003 and 2007–08 Canadian Community Health Survey (CCHS). The CCHS is a cross-sectional survey annually administered by Statistics Canada and collects self-reported health data. Briefly, the CCHS utilizes a multi-stage, stratified, clustered-probability survey sampling design that is administered to 98% of the Canadian population that is 12 years of age or older, excluding those living on reserves, individuals residing within institutions, and full-time members of the Canadian Armed Forces [22]. Details of the CCHS survey methodology are documented elsewhere [22].

Since all residents of the province of Ontario are covered by a universal, single payer insurance system known as the Ontario Health Insurance Plan (OHIP), health system encounters can be tracked. With de-identified OHIP health card numbers, health encounters are recorded in administrative health data and held at the Institute for Clinical Evaluative Sciences (ICES). The Ontario Diabetes Database (ODD) is an ICES-derived disease registry used to identify all physician-diagnosed cases of diabetes in Ontario [23]. The ODD uses the diagnostic criteria of two physician service claims recorded in OHIP or one hospital discharge related to diabetes within a two-year period to identify incident diabetes cases. The ODD has been validated with a sensitivity of 86% and a specificity of 97% for classifying individuals with or without type 2 diabetes [23]. These datasets were linked using unique encoded identifiers and analyzed at ICES.

The final study sample was restricted to CCHS participants (2003 or 2007–08) over the age of 40 years at the interview date and with a diabetes diagnosis from the ODD. Individuals who did not participate in the oral health component of the CCHS or were OHIP-ineligible for the entire observation window were excluded. The final cohort consisted of 5,183 individuals, representing a weighted sample of 1.31 million Ontario residents.

### Oral health

The oral health content of CCHS cycles 2003 and 2007–08 was selected to ensure the availability of oral health linked electronic medical records. Cycles were combined using a pooled approach to increase the sample size, providing greater statistical power [24]. Self-reported oral health status is the exposure variable and was used as a proxy for periodontal condition. It was assessed through the question: “Would you say the health of your teeth and mouth is: excellent, very good, good, fair, or poor?” Since there were low numbers of respondents in the “poor,” “fair,” and “excellent” categories, the variable was grouped into “good to excellent” versus “poor to fair” oral health groups. Further description of oral health content in the CCHS is described elsewhere [22].

### Diabetes complications

CCHS respondents with a diabetes diagnosis were followed prospectively from the survey interview date until March 31, 2016. Only the first diabetes complication following the interview date was captured from electronic medical records, and those who did not experience a diabetes complication or died before the end of the follow-up period were censored. Participants were categorized according to those who experienced a complication and those who did not. Complications were defined by select International Classification of Disease (ICD) codes previously used in the literature [25]. Complications included hyper- and hypo-glycemia, myocardial infarction, stroke, skin infection, amputation, kidney failure, dialysis and retinopathy.

### Baseline covariates

At the CCHS interview date, survey participants reported demographic characteristics, health behaviours and medical histories. Covariates from the CCHS, which were included in our analysis, were age, gender, income, education, immigrant status, race, physical activity, smoking status, alcohol consumption, dental visits, BMI, comorbidity, stress, and self-reported overall health.

Physical activity was measured using an index derived from self-reported physical activities in the past three months prior to the interview date [26]. Smoking history was derived using lifetime cigarette consumption and categorized as current smoker, former smoker and never smoked [26]. Alcohol consumption was characterized as regular, occasional, former or never had a drink in the past 12 months [26]. Stress was defined by the amount of stress perceived by study participants on most days, dichotomized into “perceiving stress” and “not perceiving stress”. Comorbidity at the interview date was evaluated by any self-reported chronic disease diagnosed by a physician other than diabetes. Participants were allocated to groups without any comorbidity or those with arthritis, COPD, heart disease or stroke at interview date. Ethnicity was categorized as “white” or “ethnic minority”, with the latter comprising Black, South Asian, Latin American, Indigenous and other ethnicities.

Other covariates extracted from electronic medical records include rural-urban dwelling, the duration of diabetes prior to the interview date, and medical care received prior to the first complication experienced. The Rurality Index of Ontario (RIO) was used to distinguish participants residing in rural areas from those in urban areas, with RIO scores of 0–39 considered urban dwelling and scores greater than 40 considered rural dwelling [27]. The duration of diabetes prior to the interview date was captured from the ODD. The type of medical care was assessed with OHIP codes for general practitioner (GP) and/or specialist visits. Medical care could be provided by a GP, specialist, or by both a GP and specialist prior to the diabetes complication.

### Statistical analysis

At baseline, all variables were assessed for their association with self-reported oral health. Baseline characteristics were compared among participants reporting “good to excellent” and “poor to fair” oral health using t-tests for continuous variables and chi-squared tests for categorical variables. Bivariate analysis was then conducted for all variables by the dichotomous outcome. A p-value of 0.25 was used as a cut-off point in the bivariate analysis in order to qualify variables for a forward building model [28].

**Fig 1:**
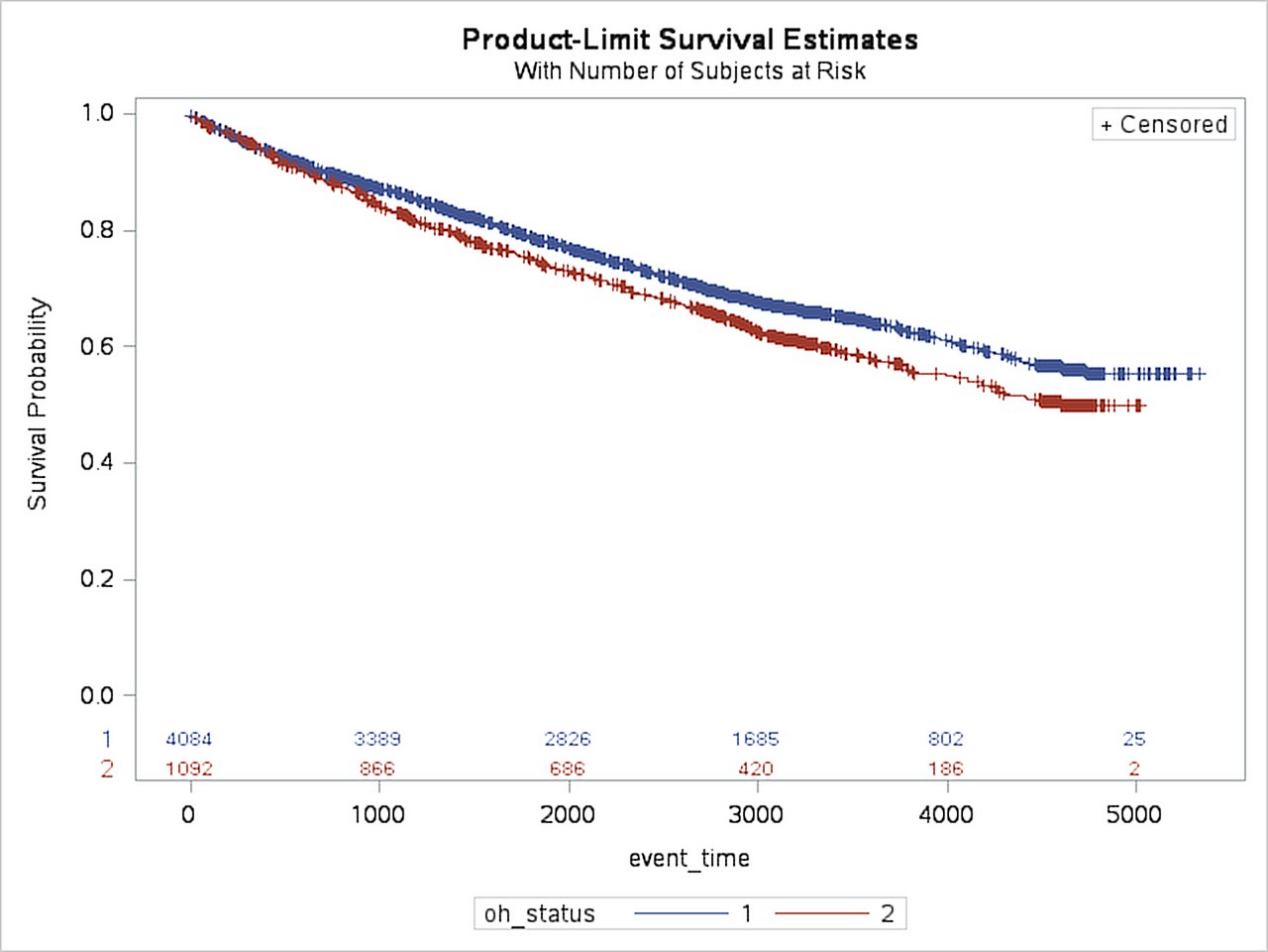
Log-rank test for probabilities of diabetes complications among study participants self-reporting “good to excellent” (oh_status = 1) and “poor to fair” (oh_status = 2) oral health status over event time in days. (n = 5,183; N = 1,308,911).

Cox proportional hazards models were built using person-days as the time-to-event for the first diabetes event. Parsimonious models were built and multivariable hazard ratios (HRs) with confidence intervals (CIs) were estimated for diabetes events associated with “poor to fair” oral health. The proportional hazard assumption was met by using the log rank test. The survival curves from the log rank test of diabetes events by self-reported oral health are shown in Fig 1. Individuals reporting “good to excellent” oral health represented the reference category.

Four primary models were constructed to test the association between oral health and diabetes complications. Following a base crude model (model 1), model 2 adjusted for age and sex. This was followed by additional adjustments for social demographic factors including income, education, ethnicity, marital status, immigrant status and RIO scores (model 3). A final adjustment in model 4 was conducted for behavioural and other influencing factors including BMI, activity index, and alcohol consumption, smoking behaviour, comorbidity, sense of community belonging and dental visits. Fully adjusted hazards were then used to estimate the population attributable risk (PAR) of complications due to “poor to fair” oral health status. As shown in the following formula [29], the prevalence of “poor to fair” oral health in the study as well as the hazards for complications was used to determine the PAR,

PAR=Prevalence*((Hazardratio‐1)/Hazardratio)

Using this estimate, the PAR of diabetes complications for the weighted Ontario population can be inferred, representing diabetics over the age of 40 who are experiencing complications that are attributable to their self-report of “poor to fair” oral health status.

Bootstrapping sample weights provided by Statistics Canada were applied to the analyses to adjust for the complex survey design of the CCHS and to generate estimates for the Ontario population. All statistical analyses were performed using SAS version 9.4 [30]. PROC SURVEY FREQ, PROC SURVEY MEANS AND PROC SURVEY PHREG were used in SAS. All CCHS respondents provided consent for Registered Persons Database (RPDB) linkage with electronic medical records during CCHS administration.

Ethical approval for this study was obtained from the University of Toronto Health Sciences Research Ethics Board followed by subsequent ICES approval for data creation and access (protocol reference #34553). Data from all databases used for this study were de-identified and no extraction of personally identifiable information occurred. There was no recruitment of any participants, no access to patient identifiers, and no contact with individuals. All administrative data received and held at ICES are used only for the approved research. Only de-identified variables specific to the study were used. ICES data cannot be copied or transferred out of ICES, except for the results of analysis, which may be removed subject to ICES approval.

## Results

The baseline characteristics of study participants according to oral health status are presented in Table 1. Participants reporting “poor to fair” oral health had a mean age of 62 years, were male and of white ethnicity. In comparison to participants with “good to excellent” oral health, those with “poor to fair” oral health were from the lowest income quintile, were less educated, reported a comorbidity prior to interview date, and had a higher mean BMI. They also reported greater “poor to fair” overall health and mental health, as well as a lower sense of community belonging. They were current smokers, living inactive lifestyles, and made fewer dental visits than those with “good to excellent” oral health.

**Table 1:** Baseline weighted characteristics of CCHS survey participants according to self-reported oral health status (n = 5,183; N = 1,308,911).

Baseline Characteristics*		“Good to Excellent” (n = 4090)	“Poor to Fair” (n = 1093)	P-value
Age (Mean, ±SD)		62.6 ± 0.4	62.2 ± 0.8	0.24
Length of follow-up (years) (Median, SD)		8.6 ± 0.1	8.3 ± 0.1	0.01
Sex (% Men)		52.2	52.2	0.993
Race (% White)		77.2	70.1	0.037
Income (%)	Quintile 1	12.5	25.4	<0.001
	Quintile2	13.4	13.9	
	Quintile 3	20.3	17.2	
	Quintile 4	21.1	16.0	
	Quintile 5	19.7	9.5	
Education (%)	< Diploma	20.2	36.58	<0.0001
	Diploma	14.5	9.81	
	Post-Secondary	60.8	49.16	
RIO (%)	Rural	90.6	92.6	0.084
	Urban	9.4	7.4	
Chronic disease (%)		51.0	63.24	0.002
BMI (Mean, SD)		27.8 ± 0.2	28.7 ± 0.5	<0.0001
Diabetes duration (years) (Mean, SD)		6.8 ± 0.1	7.1 ± 0.3	0.0201
Stress (%)		45.9	30.7	<0.001
Health status (%)	Excellent	7.2	2.3	<0.0001
	Very good	22.8	10.4	
	Good	39.4	26.4	
	Fair	21.0	39.4	
	Poor	9.5	21.5	
Mental health status (%)	Excellent	36.2	22.9	<0.0001
	Very good	33.8	26.5	
	Good	23.8	34.7	
	Fair	4.8	8.1	
	Poor	1.2	7.8	
Community belonging (%)		70.1	53.6	<0.0001
Smoking (%)	Current	12.9	20.9	0.014
	Former	48.6	42.5	
	Never smoked	38.5	36.6	
Alcohol Use (%)	Regular	47.2	35.7	0.008
	Occasionally	19.7	18.3	
	Former	29.0	38.5	
	Never drank	4.1	7.5	
Activity Index (%)	Active	19.1	13.6	0.056
	Mod. active	21.6	18.3	
	Inactive	59.3	68.1	
Dental Visits (%)	0/year	37.5	47.8	0.0071
	1–2 visits/year	46.8	35.7	
	>2 visits/year	15.7	16.5	
Pain teeth/gums (%)	Often	1.7	12.6	<0.0001
	Sometimes	11.4	29.5	
	Rarely	16.8	19.1	
	Never	70	38.9	
Physician care (%)	GP	8.9	8.0	0.0083
	Specialist	8.2	15.0	
	Concurrent	82.9	77.0	

*Chi-squared test statistic used to compare characteristics of study participants according to self-reported oral health categories. Note: percentages may not add up to 100% because of missing categories or rounding

During the observation window, 35% of all participants had a diabetes complication following the interview date (Table 2). Approximately 38% of those with “poor to fair” oral health had a diabetes complication in comparison to 34% of those with “good to excellent” oral health (Table 2).

**Table 2:** Diabetes complications experienced by participants self- reporting their oral health (n = 5,183; N = 1,308,911).

Oral Health Status	Event Type* No complication Complication		Total
“good to excellent”	2693 (65.8%)	1397 (34.2%)	4090
“fair to poor”	674 (61.7%)	419 (38.3%)	1093
Total	3367	1816	5183
p = 0.008			

*Percentages calculated are row percentages

The probability of a diabetes complication by self-reported oral health is reported in Fig 1. As shown, individuals with “poor to fair” oral health had a slightly lower survival probability over time than individuals with “good to excellent” oral health. The proportional hazard assumption is made for Cox Proportional Hazard analysis and the log rank test. This test resulted in a p-value of <0.01, supporting the hypothesis that there is a difference between study participants reporting “good to excellent” and “poor to fair” oral health.

The hazard ratios, as shown in Table 3, depict the difference in risk of diabetes complications among participants’ oral health. The age- and sex-adjusted risk of a diabetes complications among participants reporting “poor to fair” oral health was 49% greater than those reporting “good to excellent” oral health [HR 1.49; 95% CI: 1.16, 1.92]. Additional adjustment for sociodemographic characteristics including income, education, race, and rural urban dwelling, only slightly attenuated the association between diabetes complication events and oral health [HR 1.48; 95% CI: 1.18, 1.85]. Further adjusting for behavioural characteristics resulted in the greatest reduction in risk for diabetes complications. Participants with “poor to fair” oral health had a 29% greater risk of a diabetes complication event, than those with “good to excellent” oral health [HR 1.29; 95% CI: 1.03, 1.61] in the fully adjusted model.

**Table 3:** Multivariable hazard ratios for diabetes complication risk by self-reported oral health (n = 5183).

Oral Health Status	Model 1^a^	Model 2^b^	Model 3^c^	Model 4^d^
Good–Excellent (Reference Group)	1.00	1.00	1.00	1.00
Poor—Fair 95% CI	1.47 (1.15, 1.87)	1.49 (1.16,1.92)	1.48 (1.19, 1.85)	1.29 (1.03, 1.61)

a. Model 1 is the crude model

b. Model 2 adjusted for age, sex

c. Model 3 adjusted for age, sex, income, education, ethnicity, marital status, immigrant status, RIO

d. Model 4 adjusted for age, sex, income, education, ethnicity, marital status, immigrant status, RIO, BMI, activity index, alcohol use, smoking, comorbidity, dental visits

Using the fully adjusted hazard for a diabetes complication among participants reporting “poor to fair” oral health, the PAR was estimated to be 5.2% (95% CI: 0.67, 8.74), meaning that 5.2% of the complications experienced by the diabetics in our study may be attributed to a poor to fair self-report of oral health.

## Discussion and conclusion

This study’s findings indicate that self-reported oral health is associated with an increased risk of diabetes complications after adjusting for a wide range of confounders, and that poor to fair oral health may be attributed with diabetes complications at the population level. This aligns with current evidence for the association between periodontal disease and diabetes complications, such as retinopathy, neuropathy, chronic kidney disease and cardiovascular conditions [31–34]. This study also contributes to insights about the public health impact of oral health in relation to diabetes complications.

Recent studies have hypothesized that, because periodontal disease manifests as a low grade chronic inflammatory condition, it has the potential to contribute to the generation of a systemic inflammatory phenotype [35]. This hypothesis is consistent with evidence demonstrating that individuals with periodontal disease have higher markers of inflammation [4,36]. Notably, these markers are found at lower concentrations among healthy individuals [36]. This supports the concept that periodontal disease contributes independently to systemic inflammation. Yet, in contrast, a recent longitudinal study looking at the association between periodontitis and glycated haemoglobin (HbA1c) during the third and fourth decades of life found that periodontal disease markers were not associated with dysglycemia [37]. This may differ for those over the age of 40, however, and more research is arguably needed to confirm the extent of the periodontal disease contribution to diabetes and diabetes-related complications as a function of age.

This study’s findings are consistent with clinical studies showing higher systemic inflammatory markers in individuals with gingivitis than those without [38]. In particular, studies of experimental gingivitis have observed that diabetics develop a greater inflammatory response to experimental plaque accumulation [39]. Systematic reviews have also found temporary reductions in blood sugar levels among diabetics following periodontal therapy [4]. Although these reductions in blood sugar levels were minimal just after care, regularly provided periodontal therapy has the potential to improve metabolic control over longer periods of time, thereby potentially altering the inflammatory profile of individuals and reducing the risk of chronic complications.

The notion that health and health outcomes are socially patterned, may also impact the oral health-diabetes pathway. Although there is considerable evidence for the biological mechanism linking oral health and diabetes, understanding the impacts of socially-patterned lifestyles and social structures themselves on the oral-systemic link may provide more insight into the role periodontal disease plays in overall health. As such, this study conducted sequential adjustments for social factors and health behaviours in its analysis. Although the social determinants have gained popularity in public health and widespread evidence suggests that their impacts are greater than health behaviours for improving population health, the impact of health behaviours on diabetes outcomes was noteworthy in this study. Of particular interest was the minimal reduction in the hazard of complications following adjustments for income, education, immigrant status, ethnicity and rural-urban living status. By comparison, adjustments for health behaviours, including physical activity index, alcohol consumption, smoking as well as dental visits, resulted in a significant reduction in the hazard of complications. This may suggest that health behaviours play a prominent role in a common risk factor approach to oral health and diabetes and may offer guidance to the development of interventions that address both social and behavioural factors.

This study had several strengths. First, much of the current literature on oral health and diabetes has focused on clinical interventions to assess the association between periodontal disease and diabetes [4]. By contrast, this study identified diabetes complications associated with oral health at the population level. Second, a validated measure of diabetes diagnosis from the Ontario Diabetes Database (ODD) was used, whereas much of the current literature has not specified the validity of a diabetes diagnosis from insurance claims [17,18]. Our study also utilized self-reported oral health—as the exposure variable and proxy for periodontal condition. As self-reported oral health is a multi-faceted measure for overall oral condition, which includes social, psychosocial, economic and cultural components of oral health, it presents as a convenient and effective measure for inferring diabetes complication risk [40]. Notably, studies have found that self-reported oral health is concordant with the clinical need for oral treatment [41,42]. The retrospective selection of our cohort and longitudinal follow-up also presents as a significant strength because the reporting of oral health status preceded the diabetes complications, thus temporality was demonstrated. Lastly, because the CCHS has less than 2% missing data and is representative of the Ontario population, this study’s findings are generalizable to those with diabetes in Ontario.

There are important limitations to consider as well. Although self-reported oral health is an acceptable proxy for periodontal disease at the population level and for epidemiological surveys, it may not adequately measure the clinical manifestations of disease [41]. Previous studies have shown that self-reported oral health information is highly specific but not sensitive [43]. In the case of periodontal disease, individuals were able to report that they did not have periodontal disease nor did they need dental treatment with greater accuracy than those who did in fact have the condition or unmet treatment needs [43]. Although clinical measures of periodontal disease, such as gingival probing depths, would provide power to this study, such measures have not been linked to electronic health records in Canada. Having time varying accounts of self-reported oral health status would also strengthen this study’s findings. As well, factors such as diet and HbA1c levels have important impacts on the diabetes condition. In this study, diet related variables including “fruits and vegetable consumption” and “eating behaviours” were explored. However, as many participants had missing data for this optional CCHS content, diet could not be accounted for in our analysis. Similarly, our study did not compare between participants with controlled and uncontrolled diabetes. This was due to the lack of lab records available in ICES data holdings.

In regards to the inclusion criteria, although the ODD has a high sensitivity and specificity in capturing diabetic patients from electronic health records, there is still a chance that some individuals might be missing [23]. This may include individuals that have not been diagnosed with the condition or those who do not encounter the health care system regularly. However, as the prevalence of undiagnosed diabetes in Canada is approximately 1.1%, missed cases would not significantly impact this study’s findings [23]. Since the periodontal disease-diabetes link is based on the biological mechanism linked to adult onset diabetes, and the ODD does not differentiate between type 1 or type 2 diabetes, this study’s results may also be overestimated. However, as more than 95% of ODD cases are made up of individuals with type 2 diabetes and this study was restricted to adults over the age of 40, the likelihood of overestimation is reduced. Misclassification error could occur for diabetes complications utilized in this study. Although the ICD codes identifying diabetes complications were previously used in other studies, there may be some codes that were missed or others that were misclassified. Lastly, although this study was able to control for many potential confounders, there may be residual confounding that remains unaccounted for. However, due to the high quality of linked CCHS data and adjustments made for many potential confounders of oral health and diabetes complications, residual confounding was arguably minimized.

In general, our study findings have implications for improved overall health. While recent interventions addressing the periodontal disease-diabetes link have been developed at the community level, including referrals networks, diabetes screening and oral health education in various settings [44–46], a population-level intervention or policy addressing the periodontal disease-diabetes link does not exist in Canada. This may be due to the paucity of population level estimates for the impact of oral health in relation to improving diabetes related outcomes. The PAR in our study does offer a measure for the potential global impact of oral health on diabetes related outcomes, thus it can help decision makers prioritize health care strategies among the population of interest [47]. It provides an understanding of the hypothetical reduction in the incidence of diabetes complications that may be observed if diabetics residing in Ontario only reported good to excellent oral health status. Although the PAR can be highly impactful for understanding population level health, it must be interpreted with care, as it is often seen as the link between causality and public health action. More research regarding the biological underpinnings of the oral health–diabetes mechanism and their syndemic nature is needed to support the PAR and to guide the prioritization of public health interventions targeting diabetics [48,49]. Nevertheless, the current literature highlights the importance of interventions that integrate oral health into universal health care delivery models in Canada to improve overall health, and there has been some political support for this concept [50,51].

Overall, within its limits, this study’s findings show that “poor to fair” oral health status may be attributed to the health complications among diabetics, after adjusting for a wide range of confounders. This has important public health implications for diabetics in Ontario, Canada.
